# Dr. Hope on the Treatment of Chronic Rheumatism

**Published:** 1842-04-23

**Authors:** 


*Active chronic rheumatism.* Here calomel and opium may be given in smaller doses, as gr. v. of calomel and gr. j. of opium, every night; but they require to be continued for a longer time, as five or six nights. Care should, however, be taken to stop short of ptyalism, especially in the scrofulous. The other particulars of the treatment are the same as in the acute form. Local treatment, however, is more beneficial than in the latter: namely, the bleedings, if necessary, may be local instead of general, and blisters, liniments, plasters, &c. may ultimately be emyloyed if a joint continues obstinately affected.

I cannot doubt that the opium contributes importantly to the cure—perhaps by allaying pain, and thus diminishing the irritative fever dependant on it; or, possibly by modifying in some unknown way the vital constitution of the blood. However this be, I have assured myself of the fact that opiates and purging alone, will cure many cases of acute rheumatism remarkably well. Others have used different narcotics with similar sucess. My friend, Dr. Lombard of Geneva, states that he has had remarkable success with the spirituous extract of aconite, in doses of gr. half, gradually increased to gr. ij. or even iij. every three hours. I have also heard that ℈j. of conium daily, in divided doses, has produced good results.

M. Bouillaud has lately extolled, and introduced to his countrymen, apparently as a novelty, the plan of copious and frequent bleeding at short intervals for acute rheumatism. This plan, which is as old as Sydenham, and which I saw carried to its utmost limits, in Scotland, nearly twenty years ago, is not to be compared in efficacy with the plan above described, either as a prompt means of curing rheumatism, or an effectual mode of preventing inflammation of the heart; while it has the advantage of exceedingly reducing the strength, and rendering convalescence very protracted. I readily admit, however that I have seen many cases promptly and effectually cured by this plan.

